# Draft genome sequence of *Methanobrevibacter smithii* strain B181, isolated from a fecal sample

**DOI:** 10.1128/mra.00320-24

**Published:** 2024-06-12

**Authors:** Caroline M. Plugge, Maryse D. Berkhout, Anastasia Galani, Taojun Wang, Clara Belzer

**Affiliations:** 1 Laboratory of Microbiology, Wageningen University and Research, Wageningen, the Netherlands; Loyola University Chicago, Chicago, Illinois, USA

**Keywords:** methanogen, human gut, hydrogen, formate

## Abstract

A mesophilic methanogen, *Methanobrevibacter smithii* B181 (DSM 11975) was previously isolated from a human fecal sample, grown on carbon dioxide and hydrogen, and subsequently sequenced. The reconstructed 1.9-Mb genome sequence of *Methanobrevibacter smithii* B181 contributes to our understanding of hydrogenotrophic, CO_2_-reducing methanogenesis in the human gut.

## ANNOUNCEMENT

Methanogenic archaea that reduce CO_2_ with H_2_ to form methane are common inhabitants of gastrointestinal tract ecosystems, with *Methanobrevibacter smithii* as the most predominant species (1–3). Although several strains have been isolated from gut ecosystems, their role in health and disease is still understudied (3–5).

During an investigation, a synthetic community approach was used to study the nutrient-driven ecological interactions occurring in a complex mucosal environment. The focus was on revealing interactions between mucin glycans and a selection of well-characterized human gut microbes, including *Methanobrevibacter smithii* strain B181 (DSM 11975). *M. smithii* strain B181 was obtained from the DSMZ culture collection. The strain was grown under strict anoxic conditions for 72 hours at 37°C in 120 mL serum bottles with 50 mL of bicarbonate-buffered medium as described previously (6), enriched per liter with 60 g brain heart infusion, 60 g proteose peptone, 10 g yeast extract, 10 g sodium acetate, and 20 g sodium formate. Here, we report the complete genome sequence of this human gut methanogen.

The genomic DNA of *M. smithii* was extracted using the FastDNA SPIN Kit for Soil (MP Biomedicals) according to the manufacturer’s instructions. Novogene NGS DNA Library Prep Set (Cat No.PT004)* is used for library preparation. Index codes were added to each sample. Briefly, the genomic DNA is randomly fragmented to a size of 350 bp. DNA fragments were end polished, A-tailed, ligated with adapters, size selected, and further Rolling Circle Amplification. Then PCR products were purified (AMPure XP system), followed by size distribution by Agilent 2100 Bioanalyzer (Agilent Technologies, CA, USA), and quantified using real-time PCR. The library was then sequenced on Illumina NovaSeq 6000 S4 flowcell with PE150 strategy and generated 41188428 raw reads.

The quality of the raw data was assessed with FastQC v0.11.9 (7). Sequencing adapters and phiX contamination were removed with the bbduk.sh script from BBtools v38.84 (8). An assembly was performed using SPAdes v3.15.2 (9), with the isolate parameter enabled from 20,593,234 (20.6 Gb) quality-filtered reads. Contigs shorter than 1,000 bp were removed with the reformat.sh script from BBTools v38.84. The quality of the resulting assembly was assessed with QUAST v5.0.2 (10). Completeness and contamination values were calculated with CheckM v1.0.18 (11).

Mapping was done with Bowtie2 v2.4.1 (12), resulting in a SAM file. This SAM file was converted to a BAM file, sorted, and indexed with SAMtools v1.18 (13). The genomecov script of BedTools v2.29.1 (14) was used to calculate the genome coverage. The genome was annotated using the NCBI Prokaryotic Genome Annotation Pipeline version 6.7 (15), upon submission to NCBI. All programs were run with default parameters. The assembled genome had a total length of 1,851,101 bp with a GC content of 31.03%. The genome coverage was 3306.38. The final assembly consisted of 20 contigs, and the N50 value was 199,244. The genome was 100% complete and 0% contaminated.

Strain B181 genome contains 1,766 protein-coding genes and 42 RNA-coding genes [34 tRNAs, 1 5S rRNA, 2 16S rRNAs, 3 23S rRNAs, and 2 non-coding rRNAs (ncRNAs)].

Strain B181 genome encodes two putative formate dehydrogenases and a putative formate transporter, suggesting that formate can also be used as a substrate. Besides, several hydrogenases, including F420 reducing hydrogenase, and membrane-associated energy-converting hydrogenases.

## Data Availability

The draft genome sequence was deposited at DDBJ/ENA/GenBank under the accession JAXOJW000000000 *via* BioProject PRJNA1051000 (with BioSample accession SAMN38759361, SRA accession SRX23002010).
